# A new strategy for monitoring of direct oral anticoagulants in patients with cyanotic and complex congenital heart disease

**DOI:** 10.1016/j.ijcchd.2024.100545

**Published:** 2024-09-25

**Authors:** Fabienne Dirbach, Eleni Goulouti, Judith Bouchardy, Magalie Ladouceur, Lorenzo Alberio, Tobias Rutz

**Affiliations:** a Service of Cardiology, Lausanne University Hospital and University of Lausanne, Lausanne, Switzerland; b Department of Cardiology, Inselspital, Bern University Hospital, University of Bern, Bern, Switzerland; c Service of Hematology and Central Hematology Laboratory, Lausanne University Hospital and University of Lausanne, Lausanne, Switzerland

**Keywords:** Congenital heart disease, Atrial arrhythmias, Anticoagulation, Direct oral anticoagulant, Vitamin K antagonist, D-dimers, DOAC

## Abstract

**Background:**

Patients with congenital heart disease (CHD) often require an oral anticoagulation. Vitamin K antagonists (VKA) are the standard treatment, however, an increased hematocrit in patients with secondary erythrocytosis due to cyanosis complicates the correct measurement of the international normalized ratio. Direct oral anticoagulants (DOAC) could be an alternative, but data on their efficacy and safety in complex and cyanotic CHD patients are scarce. This study proposes a new strategy of DOAC monitoring in these patients using D-dimers and DOAC trough levels.

**Methods:**

This is a retrospective study including cyanotic and complex CHD patients requiring oral anticoagulation. Clinical, cardiac imaging and laboratory data were collected before and after start of DOAC. The new monitoring strategy consists of determination of D-dimers and DOAC trough levels at 1–4 weeks, 1–6 months, 6–12 months, >1 year after start of DOAC.

**Results:**

Eleven patients were included. For 10 patients D-dimers and DOAC trough levels were in target range. In one patient, D-dimers increased continuously after start of DOAC despite dose escalation, suggesting insufficient DOAC efficacy and finally requiring a switch to VKA. D-dimers subsequently decreased under VKA to the therapeutic range. In three patients, one thromboembolic and two minor bleeding complications occurred. No major complications were observed.

**Conclusions:**

We propose a new strategy of monitoring of oral anticoagulation with DOAC and report its implementation in clinical routine. Highlighting the importance of pharmacokinetic and -dynamic monitoring, this strategy could improve safety and efficacy of DOAC in cyanotic and complex CHD which, however, requires a prospective validation.

## Highlights

• A new strategy of monitoring of direct oral anticoagulants (DOAC) in cyanotic and complex congenital heart disease is proposed.
• The implementation in the clinical routine of the monitoring strategy with the help of D-dimers and trough levels of DOAC is reported.
• The new strategy helps to identify an insufficient anticoagulation and to avoid potentially catastrophic events.

## 1 Introduction

Patients with congenital heart disease (CHD) are at a high risk to develop atrial arrhythmias (AA) increasing importantly their morbidity and mortality [1].

Due to an increased risk of thromboembolic (TE) events, indications for long term oral anticoagulation are frequently encountered in these patients [2]. Vitamin-K-antagonists (VKA) are considered the standard treatment requiring strict monitoring of the international normalized ratio (INR) [2]. However, cyanosis in CHD patients causes (patho-) physiological adaptations to improve oxygen transport leading to secondary erythrocytosis and elevated hematocrit (HCT) [2]. In these circumstances, an accurate determination of INR requires a manual adaptation of citrate volume of the blood drawing tubes in order to compensate for the HCT increment. This complicates the control of the INR and increases the probability of an erroneous INR [[3], [4], [5]].

An often concomitant platelet dysfunction adds to the bleeding risk in these patients [6]. At the same time, CHA_2_DS_2_-VASc and HAS-BLED scores have an uncertain validity rendering anticoagulation management especially challenging [2,7].

In the general population, direct oral anticoagulants (DOAC) are proven to be a safe and effective alternative to VKA [[8], [9], [10], [11]]. However, studies in CHD patients show conflicting results and current recommendations are mainly based on expert opinions [[12], [13], [14], [15]].

New concepts and strategies for the evaluation of the efficacy and safety of DOAC in patients with cyanotic CHD are therefore urgently required. Moner-Banet et al. discuss data suggesting that monitoring DOAC levels (pharmacokinetics) and D-dimers (pharmacodynamics) may improve anticoagulation safety and efficacy in the general population [16]. Evolution of D-dimers after start of anticoagulation can be used for pharmacodynamic monitoring of anticoagulation efficacy [[17], [18], [19], [20]]. However, until now, there are no specific data available regarding monitoring of DOAC in CHD patients.

The aims of the study were to explore the feasibility of such a new strategy of anticoagulation monitoring and its implementation in clinical routine for treatment decision-making in a complex population.

## 2 Material and methods

### 2.1 Study population

Adult CHD patients (≥18y/o) with cyanotic and/or complex CHD and oral anticoagulation with DOAC were included in this retrospective and observational study.

Patients had been previously identified by the treating cardiologist potentially being eligible for a DOAC therapy. The patients’ characteristics were evaluated together with the haematologist (LA) before start of DOAC. Usually, an individualized treatment plan was established with determination of practicable timepoints of control of anticoagulation.

Clinical, imaging and laboratory data were reviewed in a time range between 6 months before start of DOAC and their most recent follow-up (study period 2015–2023).

Clinical variables, such as type of CHD, data on cardiovascular surgery or percutaneous interventions, renal function, NYHA class, oxygen saturation and HCT were identified, as well as secondary diagnoses, and medical treatment. Oral anticoagulation indications, bleeding and/or TE complications were collected. CHA_2_DS_2_-VASc and HASBLED scores were calculated [21].

### 2.2 Assessment of anticoagulation

Anticoagulation was assessed in close collaboration between cardiologists and hematologists. Before start of DOAC, a comprehensive laboratory work-up was performed with measurements of INR, D-dimers, DOAC blank values, as well as renal and liver function tests. After start of DOAC, measures of DOAC and D-dimers were repeated usually at 1–4 weeks, 1–6 months, 6–12 months, >1 year after start of DOAC. If needed, tube's citrate volume was adapted according to the hematocrit for coagulation studies. Trough levels of DOAC were assessed with the BIOPHEN™ Heparin LRT method (HYPHEN BioMed, Neuville-sur-Oise, France), a one-stage chromogenic assay that upon specific calibration determines the concentrations of heparins or DOAC with anti-factor Xa activity by measuring the inhibition of an exogenous amount of factor-Xa, with the residual activity being indicated by a chromogenic factor Xa substrate; thus, the amount of color change is inversely proportional to the anticoagulant concentration in the sample. D-dimers were detected quantitatively with the INNOVANCE® D-dimer reagent (Siemens Healthineers, Erlangen, Germany) in a procedure using polystyrene particles coated with a monoclonal antibody causing aggregation of the particles in the sample. The concentration of D-dimers is then measured by turbidimetry of the solution. A clear therapeutic range of DOAC trough levels has not been defined until now, however, Moner-Banet et al., based on the published evidence, have identified an estimated target range, which we used as a point of reference in this study [16]. D-dimer values < 500 ng/mL were considered as a target cut-off for an effective anticoagulation (see e.g., Fig. 1 of Beyer-Westendorf et al.) [22].

**Fig. 1:**
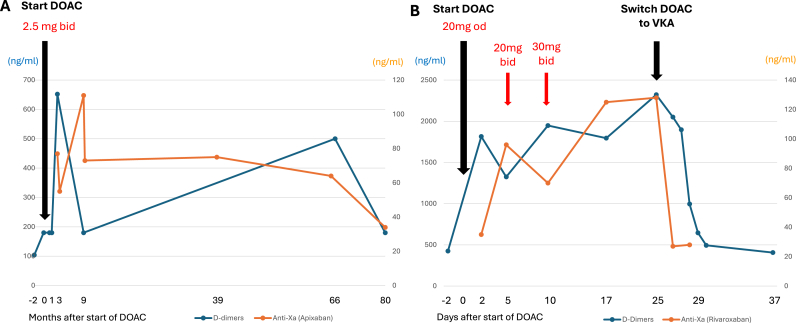
A Example of monitoring of D-dimers and DOAC trough levels of a patient over a duration of 80 months, demonstrating stability of target levels on the long term. B Example of a patient in whom D-dimers increased after introduction of DOAC despite increase of dose of rivaroxaban, while DOAC trough levels increased concomitantly, requiring ultimately replacement of DOAC by VKA, resulting in normalisation of D-dimers.

### 2.3 Endpoints

Endpoints were D-dimer elevation, stop of DOAC therapy, major and minor bleeding or TE events [21]. Major bleeding was defined based on the International Society on Thrombosis and Haemostasis (ISTH) classification as: intracranial, intraspinal, intramuscular or intraarticular bleeding, bleeding requiring transfusion, or fatal bleeding [21]. Every other type of bleeding was defined as minor. We considered the following events as TE complications: cerebrovascular accident, deep vein thrombosis, superficial vein thrombosis, pulmonary thromboembolism, thrombosis of intra- and extracardiac shunt, myocardial infarction, and other systemic thrombosis.

## 3 Results

Eleven adult (≥18 y/o) patients (mean age 38 y/o, range 19–60 y/o) with cyanotic and complex CHD were included. Clinical and laboratory baseline characteristics of the population are shown in Table 1, results of imaging exams are shown in the supplementary material (S1).

**Table 1:** Patients’ characteristics.

Patient number	Age	Sex	Diagnosis	Cardiac intervention	Co-morbidity	Drugs	Creatinine clearance (ml/min)	NYHA	Oxygen saturation	PAH	HCT	Living/deceased
1	30	M	Non corrected univentricular heart	Banding of pulmonary artery	Amiodarone induced Hyperthyroidism	Betablocker, Amiodarone	50	3	88 %	Y	56 %	Living
2	33	F	Non corrected univentricular heart	N	Cowden syndrome, liver cirrhosis (Child-Pugh B) of cardiac origin, factor II, VII, X deficiency	ACE-inhibitor/AT-1 inhibitor, Betablocker, Levothyroxine	135	3	70 %	N	66 %	Deceased
3	48	M	Palliated univentricular heart	Glenn, Blalock-Taussig shunt	Diabetes type 2	ACE-inhibitor/AT-1 inhibitor, Betablocker, Diuretics	104	3	79 %	N	68 %	Living
4	21	M	Fontan circulation	Banding of pulmonary artery, Glenn, Fontan	Hepatic fibrosis, factor VII deficiency	Betablocker	148	2	95 %	N	43 %	Living
5	60	F	Eisenmenger syndrome	N	Amiodarone induced Hyperthyroidism	Amiodarone, Spironolactone, Macitentan, Sildenafil, Diuretics	79	3	85 %	Y	50 %	Deceased
6	28	F	Palliated univentricular heart	Glenn, Blalock-Taussig shunt	N	ACE-inhibitor/AT-1 inhibitor	86	2	80 %	N	61 %	Living
7	54	F	ASD type ostium secundum	Partial closure of ASD with Amplatzer Occluder	COPD GOLD 1, Scheuermann's Kyphosis, Hashimoto's disease, femoral arteriovenous fistula	ACE-inhibitor/AT-1 inhibitor, Amiodarone, Macitentan, Sildenafil, Diuretics, Levothyroxine	71	3	91 %	Y	42 %	Living
8	43	F	TGA, situs inversus	Atrial Switch	N	ACE-inhibitor/AT-1 inhibitor	127	1	99 %	N	33 %	Living
9	38	M	Fontan circulation	Fontan, Blalock-Taussig, Glenn	Focal epilepsy, Normal pressure hydrocephalus	ACE-inhibitor/AT-1 inhibitor, Diuretics	122	2	92 %	N	54 %	Living
10	47	F	ASD type ostium secundum	Tricuspid annuloplasty, ASD closure	Status post multiples pulmonary embolisms	Betablocker, Spironolactone, Macitentan, Sildenafil, Diuretics	175	3	84 %	Y	38 %	Living
11	19	F	Non corrected univentricular heart	N	Athyreosis, thoracolumbar scoliosis	ACE-inhibitor/AT-1 inhibitor, Spironolactone, Levothyroxine, Diuretics	72	2	82 %	N	41 %^a^	Living

**Abbreviations:** AFib = atrial fibrillation, AFlut = atrial flutter, DOAC = direct oral anticoagulant, IART = intra-atrial re-entrant tachycardia, LMWH = low molecular weight heparin, OAC = oral anticoagulation, TE = thromboembolic, UFH = unfractionated heparin, VKA = vitamin K antagonist, others see Table 1.

a Patient 11 presented a low hematocrit despite a central cyanosis. The hematological work-up could not identify a cause.

Indications for oral anticoagulation were atrial fibrillation/flutter and intraatrial arrythmia in nine patients as well as deep vein thrombosis, suspicion of Glenn thrombosis, ischemic vascular accident in one, respectively, with one patient fulfilling two indications. Table 2 provides detailed information on oral anticoagulation therapy for each respective patient.

**Table 2:** Indications and information on oral anticoagulation.

Patient number	Reason for anticoagulation	CHA2DS2-VASc score	HASBLED score	Reason for switch to DOAC	Type of DOAC	Anticoagulation drug before DOAC	Thromboembolic complications under DOAC	Bleeding complications under DOAC	Duration of DOAC treatment
1	AFib	2	2	INR lability	Apixaban	Acenocoumarol	Superficial venous thrombosis	N	6.6 years
2	Deep vein thrombosis, AFib	4	5	De novo introduction, existence of antidote of Dabigatran	Dabigatran	UFH	N	Cutaneous hematoma	3 months
3	Suspicion of shunt Glenn thrombosis	4	3	Hyperkalemia under LMWH	Rivaroxaban	LMWH	N	N	25 days
4	Recurring supraventricular tachycardias	0	1	Incompliance with VKA	Rivaroxaban	Acenocoumarol	N	N	3 years
5	AFib	1	1	De novo introduction	Apixaban	UFH	N	N	3 years
6	Ischemic vascular accident	3	1	Treatment simplification	Apixaban	Acenocoumarol	N	N	3 years
7	AFib and AFlut	0	0	Minor risk of cerebral hemorrhage, treatment simplification	Apixaban	Acenocoumarol	N	N	2 years
8	IART and AFlut	1	1	Treatment simplification	Apixaban	Acenocoumarol	N	Epistaxis	7.5 years
9	AFlut	0	0	Treatment simplification	Rivaroxaban	Acenocoumarol	N	N	1.5 years
10	AFlut	5	0	Treatment simplification	Apixaban	Acenocoumarol	N	N	8 months
11	AFib	1	0	De novo introduction	Apixaban	LMWH	N	N	3 months

**Abbreviations:** AFib = atrial fibrillation, AFlut = atrial flutter, DOAC = direct oral anticoagulant, IART = intra-atrial re-entrant tachycardia, LMWH = low molecular weight heparin, OAC = oral anticoagulation, TE = thromboembolic, UFH = unfractionated heparin, VKA = vitamin K antagonist, others see Table 1.

Before start of DOAC, median D-dimer levels of the total patient population were 303 ng/ml (IQR 180–604), INR for the patients on VKA 2.2 (IQR 1–3), platelets 192 G/l (IQR 131–262), and NT-proBNP 749 ng/l (IQR 589–1368). The median HCT of the seven patients with central cyanosis was 56 % (IQR 41–66) while the median HCT of the four non-cyanotic patients was 42.5 % (IQR 38–43). Mean duration of DOAC therapy was 31 ± 29 months (range 1–92 months). Ten patients were treated with a factor Xa inhibitor, one patient with factor IIa inhibitor (Table 2).

### 3.1 Follow-up of anticoagulation

Table 3 indicates the evolution of D-dimers, INR, and trough levels before and after DOAC start. In 10 out of the 11 included patients, D-dimers and trough levels were in target range and oral anticoagulation with DOAC was continued without major clinical complications. Figure A shows the example of the monitoring of D-dimers and DOAC trough levels of a patient over a total duration of 80 months. In patient three, in contrast (Figure B), D-dimers increased from 425 ng/ml to 2322 ng/ml within 25 days after introduction of rivaroxaban despite progressive dose escalation to 2x30 mg/day and concomitant rise of trough levels to 128 ng/ml. Anticoagulation by DOAC was therefore considered as inefficient and rivaroxaban was stopped after 25 days. VKA were started concomitantly with a therapeutic anticoagulation with unfractionated heparin. D-dimers decreased to normal values under VKA and heparin (494 ng/ml, anti-Xa activity heparin 0.4 U anti-Xa/ml). After reaching the target INR of 2–3 twice on 24 h apart, heparin was stopped. D-Dimers remained within target range (<500 ng/ml) at 6 weeks (INR 2.9, D-dimers 406 ng/ml) and were suppressed at 9 months (<190 ng/ml, Table 3).

**Table 3:** Hematological data during follow-up.

	Before start of DOAC			1–4 weeks after start of DOAC			1–6 months after start of DOAC			6–12 months after start of DOAC			>1 year after start of DOAC		
Patient number	INR	Anti-Xa activity heparin (U anti-Xa/ml)	D-dimers (ng/ml)	INR	DOAC trough level (ng/ml)	D-dimers (ng/ml)	INR	DOAC trough level (ng/ml)	D-dimers (ng/ml)	INR	DOAC trough level (ng/ml)	D-dimers (ng/ml)	INR	DOAC trough level (ng/ml)	D-dimers (ng/ml)
**1**	2.2		<190				1.1	77	652	1.3	111	<190	1.2	64	500
**2**	1.5	0.4	1874	1.5	57	543		109							
**3**	1	0.55	425	1.3	128	2322	1.1 (Acenocoumarol and heparin) ∗		494	2.6		<190	3.5 (Acenocoumarol)		283
**4**	1.2		<190	1.3	64	<190				1.4			1.3		
**5**	1.1	0.45	521		174	213				1.3			1.2	317	
**6**	2.2		<190		95	<190									
**7**	3						1.1			1.1	220	<190			
**8**	1												1	89	
**9**	4						1.2	35	<190	1.7	287		1.5	142	<190
**10**	1			1.1			1	88	<190						
**11**	1.2	0.3	664	1.1	178	3573	1.2	206	1560			880			

**Abbreviations:** INR = international normalized ratio, others see Table 1, Table 2, ∗Anti-Xa activity of heparin: 0.4 U anti-Xa/ml.

Note that the initial increase of D-dimers in patient 11 was related to an intercurrent infection. D-dimers subsequently decreased with the resolution of the infection.

### 3.2 Complications under DOAC

No major TE or bleeding complications were observed. One patient (9 %) suffered a minor TE complication (a superficial venous thrombosis that occurred 16 months after introduction of DOAC). Two patients (18 %) had minor bleeding complications (a cutaneous hematoma four days, and an epistaxis five days after introduction of DOAC). No complication required stop or change of DOAC. The patient showing increase of D-dimers and requiring switch to VKA treatment did not present any TE or bleeding complications under DOAC. However, one year after switch back to VKA, this patient suffered from a stroke of cardio-embolic origin due to atrial fibrillation and subtherapeutic INR (1.8).

Two patients deceased during follow-up with death not related to oral anticoagulation: one end stage CHD with Eisenmenger syndrome, one respiratory infection.

## 4 Discussion

In the present study, we describe the implementation of a new strategy for DOAC monitoring in cyanotic and complex CHD.

### 4.1 Monitoring strategy

Based on previous reports in different patient populations including atrial fibrillation, cancer, obstetrics and valvular prosthesis, we used drugs’ trough levels and D-dimers values for the combined pharmacokinetic/pharmacodynamic anticoagulation follow-up after start of DOAC [[16], [17], [18], [19], [20],^23^]. In general, before treatment start with DOAC, a coagulation work-up with determination of baseline levels of D-dimers was performed. To evaluate pharmacokinetics of DOAC, we measured trough levels, providing an accurate quantitative assessment of their concentration before next administration, which has been related both to safety and efficacy [16]. Furthermore, for evaluating anticoagulation efficacy (i.e., pharmacodynamics), we tracked the evolution of D-dimers during follow-up. Studies suggest that a decrease in D-dimer plasma levels during oral anticoagulation treatment correlates with a reduced clotting activity and therefore a lower risk of TE events, demonstrating the utility of D-dimers as a marker of efficacy of oral anticoagulation in various patient populations, such as those with atrial fibrillation, thrombocytopenia or mechanical valve prostheses [16,18,22,24].

We can demonstrate that the implementation of the strategy is feasible in the clinical routine, however, requiring a close collaboration with the hematologists.

### 4.2 Complications

By applying this concept, we detected that in one patient, D-dimers continued to increase after start of DOAC despite the increase of the dose of rivaroxaban, suggesting insufficient DOAC efficacy. The concomitant increase of the trough level in parallel to the dose escalation and the fact that the patient was hospitalized, suggested that non-compliance was unlikely to be the cause for the insufficient response to DOAC. Therefore, 25 days after initiation of DOAC, a switch to VKA was performed with a good biological response (decrease of D-dimers to a target value < 500 ng/ml). No TE or bleeding complications occurred until one year later, when the patient presented a stroke under VKA due to atrial fibrillation with a subtherapeutic INR of 1.8. This event highlights the importance of strict surveillance of oral anticoagulation in this very fragile patient group.

In ten patients, monitoring showed that D-dimers and trough levels of DOAC were in target range demonstrating efficient oral anticoagulation. In three patients, two minor bleeding and a superficial TE complication occurred, without the need to interrupt or change DOAC therapy. However, and more importantly, no major complications were observed during follow-up. Our results are in line with previous studies showing that cyanotic and complex CHD patients are at high risk for TE and bleeding events [11,15,[25], [26], [27], [28]]. A high complexity of the underlying CHD lesion, unclosed septal defects, residual shunts, cyanosis and Eisenmenger syndrome are associated with a higher rate of TE events [7,[29], [30], [31], [32]]. However, our small patient population and the relatively short follow-up do not allow to draw conclusions on safety and efficacy of DOAC in the study patient population.

### 4.3 Patients with cyanotic CHD

Seven patients presented with central cyanosis. The guidelines consider VKA as the oral anticoagulation treatment of choice in cyanotic CHD with AA, due to a lack of data supporting the use of DOAC in these patients [2]. However, cyanosis causes (patho-) physiological adaptations to improve oxygen transport and delivery to the tissues, including increased erythropoietin stimulus leading to secondary erythrocytosis which results in elevated HCT and reduced plasma volume [5,6]. The latter impairs the correct measurement of the INR, an essential element in monitoring VKA levels and controlling its efficacy and safety [5,6]. Therefore, manual adaptation of the citrate volume in the blood drawing tube is required, rendering oral anticoagulation with VKA especially challenging [2].

Furthermore, due to hematological abnormalities such as impaired platelet function and altered coagulation mechanisms, patients with cyanotic CHD are at an increased risk for both thrombotic and bleeding complications [5,6]. Two cyanotic patients showed minor TE, confirming the particular vulnerability of this subgroup. No interruption or adaptation of DOAC was necessary while monitoring of trough levels of DOAC and D-dimers remained in target range during follow-up.

### 4.4 Patients with Fontan circulation

Due to blood stasis in the Fontan circulation and altered coagulation parameters, Fontan patients are at a particular high risk for TE complications as shown in the NOTE registry, in which 50 % of all TE and bleeding events occurred in patients with Fontan circulation [15]. For this reason, current guidelines propose VKA as the oral anticoagulation treatment of choice [2]. Nonetheless, several studies reported that DOAC appear to be safe in Fontan patients with rates of TE and bleeding complications comparable to VKA [25,27,33]. Our strategy could help to improve monitoring of DOAC in this patient group.

### 4.5 Limitations

This retrospective study is limited by a small and heterogenous cohort including different subgroups of complex CHD over a relatively short study period. Timing of laboratory work-up and duration of follow-up varied between patients and data were missing for some patients. Therefore, efficacy and safety of DOAC in patients with cyanotic and complex CHD cannot be evaluated with these data. In addition, no information on adherence of oral anticoagulation treatment was available. Therefore, this study is merely ought to serve as a springboard for larger and prospective studies to further evaluate the use of a combined pharmacokinetic/pharmacodynamic monitoring of DOAC in patients with complex and cyanotic CHD. We hypothesize that this approach will improve efficacy and safety of oral anticoagulation in this fragile group of patients.

## 5 Conclusion

The present study describes the development and implementation of a new concept of combined pharmacokinetic/pharmacodynamic monitoring of DOAC in patients with complex and cyanotic CHD. The proposed strategy could potentially help to improve the safety and efficacy of DOAC in these patients. However, prospective and larger studies are needed for its further validation.

## Ethics statement

The local ethics committee approved the project (CER-VD 2021–02363).

## Patient consent statement

Need for patient consent was waived by the ethics committee.

## Availability of data and materials

The datasets used and/or analyzed during the current study are available from the corresponding author on reasonable request.

## Disclosures

The authors declare no conflict of interest. Dr. Ladouceur is an Editorial Board Member of the International Journal of Cardiology Congenital Heart Disease and played no role in the Journal's evaluation of the manuscript”.

## Funding sources

This research did not receive any specific grant from funding agencies in the public, commercial, or not-for-profit sectors.

## CRediT authorship contribution statement

**Fabienne Dirbach:** Writing – review & editing, Writing – original draft, Methodology, Formal analysis, Data curation. **Eleni Goulouti:** Writing – review & editing, Data curation. **Judith Bouchardy:** Writing – review & editing. **Magalie Ladouceur:** Writing – review & editing. **Lorenzo Alberio:** Writing – review & editing, Writing – original draft, Supervision, Investigation, Conceptualization. **Tobias Rutz:** Writing – review & editing, Writing – original draft, Validation, Supervision, Resources, Project administration, Conceptualization.

## Declaration of competing interest

The authors declare that they have no known competing financial interests or personal relationships that could have appeared to influence the work reported in this paper.
