# Supplementary material for: A new strategy for monitoring of direct oral anticoagulants in patients with cyanotic and complex congenital heart disease
Source: Int J Cardiol Congenit Heart Dis. 2024 Sep 25;18:100545. doi: 10.1016/j.ijcchd.2024.100545 (PMC11657253; doi:10.1016/j.ijcchd.2024.100545)
Supplement: Multimedia component 1 [file mmc1.docx]

## Supplemental table 1

### Results of TTE and CMR before start of DOAC

| Transthoracic echocardiography | | | | | | | | Cardiac magnetic resonance | | |
| --- | --- | --- | --- | --- | --- | --- | --- | --- | --- | --- |
| Patient number | **Systemic ventricular ejection fraction (%)** | **Systemic ventricular dilatation** | **Pulmonary stenosis** | **Tricuspid stenosis** | **Tricuspid regurgitation** | **VSD** | **ASD** | **Systemic ventricular ejection fraction (%)** | **Systemic ventricular end**  **diastolic volume (mL/m2)** | **Shunt quantification (Qp : Qs)** |
| 1 | 55 | Y | Severe | Atresia | Atresia | Y | Y | 52 | 223 | 4 |
| 2 | 55 | Y | Severe | Atresia | Atresia | Y | Y | 43 | 278 | 0.38 |
| 3 | 30 | Y | Severe | Atresia | Atresia | Y | Y | 29 | 158 | 0.5 |
| 4 | 44 | N | N | N | Mild | N | N | 45 | 100 | 1.25 |
| 5 | 65 | N | Atresia | N | Mild | Y | N | 55 | 90 | 1.45 |
| 6 | 60 | Y | N | N | Moderate-severe | Y | Y | 40 | 60 | 0.47 |
| 7 | 60 | N | N | N | N | N | Y | 64 | 63 | 1.3 |
| 8 | 55 | N | N | N | Moderate | N | N | 40 | 72 | No shunt |
| 9 | 55 | N | N | Atresia | Atresia | N | N | 60 | 114 | No shunt |
| 10 | 55 | N | N | N | Severe | N | N | 57 | 87 | 1.14 |
| 11 | 55 | Y | Atresia | Atresia | Atresia | Y | Y | 56 | 260 | 2.1 |

**Abbreviations:** ASD = atrial septal defect, CMR = cardiac magnetic resonance, TTE = transthoracic echocardiography, VSD = ventricular septal defect
